# Measurement of structural integrity of the spinal cord in patients with amyotrophic lateral sclerosis using diffusion tensor magnetic resonance imaging

**DOI:** 10.1371/journal.pone.0224078

**Published:** 2019-10-29

**Authors:** Maximilian Patzig, Katja Bochmann, Jürgen Lutz, Robert Stahl, Clemens Küpper, Thomas Liebig, Peter Reilich, Marianne Dieterich

**Affiliations:** 1 Institut für Diagnostische und Interventionelle Neuroradiologie, Ludwig-Maximilians-Universität München, München, Germany; 2 Radiologisches Zentrum München Pasing, München, Germany; 3 Neurologische Klinik und Poliklinik mit Friedrich-Baur-Institut, Ludwig-Maximilians-Universität München, München, Germany; 4 Cluster for Systems Neurology, SyNergy, München, Germany; clinical research on neurological diseases, CHINA

## Abstract

**Background:**

The value of conventional magnetic resonance imaging (MRI) for amyotrophic lateral sclerosis (ALS) is low. Functional and quantitative MRI could be more accurate. We aimed to examine the value of diffusion tensor imaging (DTI) with fractional anisotropy (FA) measurements of the cervical and upper thoracic spinal cord in patients with ALS.

**Patients and methods:**

Fourteen patients with ALS and 15 sex- and age-matched controls were examined with DTI at a 3T MRI scanner. Region-of-interest (ROI) based fractional anisotropy measurements were performed at the levels C2-C4, C5-C7 and Th1-Th3. ROIs were placed at different anatomical locations of the axial cross sections of the spinal cord.

**Results:**

FA was significantly reduced in ALS patients in anterolateral ROIs and the whole cross section at the C2-C4 level and the cross section of the Th1-Th3 level. There was a trend towards a statistically significant FA reduction in the anterolateral ROIs at the C5-C7 level in ALS patients. No significant differences between patients and controls were found in posterior ROIs.

**Conclusion:**

FA was reduced in ROIs representing the motor tracts in ALS patients. DTI with FA measurements is a promising method in this circumstance. However, for DTI to become a valuable and established method in the diagnostic workup of ALS, larger studies and further standardisation are warranted.

## Introduction

Amyotrophic lateral sclerosis (ALS) is an adult onset disorder characterised by progressive degeneration of the upper and lower motor neurons with increasing atrophy and functional motor impairment following a distinct spreading pattern within the CNS [1]. Patients usually die within the frame of a ventilatory insufficiency based on a dyspnoea. This disease group is divided into two groups depending on the focus of the motor lesion (upper motor neuron in the gyrus praecentralis, lower motor neuron in the brain stem and anterior horn of the spinal cord), age at initial manifestation, the course of the disease, the initial affected muscles (bulbar, upper extremities, thoracic, lower extremities), the clinical instrumental participation of other systems (e.g. frontotemporal dementia) and etiological factors (sporadic, hereditary) in subtypes and variants. Its aetiology is still unknown. Although there is no cure for ALS as yet, an early and definite diagnosis of the disease is essential regarding patient guidance and the initiation of supportive and/or palliative care.

According to the revised El Escorial [2] and Awaji [3] criteria, the diagnosis is mainly based on clinical parameters, supported by laboratory and neurophysiological findings. Until now, imaging has a minor role in the diagnosis of ALS and is mainly used to exclude competing aetiologies such as cervical spinal canal stenosis. ALS findings on conventional MRI include motor cortex atrophy and increased T2/FLAIR signal intensity of corticospinal tracts. However, the diagnostic accuracy of such findings is low [4, 5]. Quantitative and functional imaging methods seem more promising, and several studies on voxel- or surface-based morphometry [6, 7], spectroscopy [8–10], and diffusion tensor imaging (DTI) have been conducted. Because ALS is primarily a disease of the motor tracts, DTI is a particularly promising technique, as it is able to visualise and measure the structural integrity of fibre tracts. DTI of the spinal cord is challenging, and studies in ALS patients have so far mainly focussed on the brain [9, 11–13]. However, involvement of the spinal upper motor neuron and the anterior horn cells are pivotal features of the disease. Therefore we aimed to determine the ability of DTI to measure structural damage of the spinal motor tracts in ALS patients.

## Patients and methods

The study was approved by the institutional review board (ethics committee of the medical faculty of the Ludwig-Maximilians-University Munich, IRB number 094–10), and written informed consent was obtained from all patients and controls.

Patients with definite or probable ALS according to the 2000 revised El Escorial criteria [2] were eligible for this study. Exclusion criteria were concomitant neurodegenerative diseases, spinal canal stenosis and/or any intramedullary lesion on MRI, relevant contraindications for MRI and motion artifacts which prevented a valid DTI analysis. The first fourteen patients meeting the criteria and agreeing to participate in the study were enrolled (three female, eleven male, mean age 56 years, range 39 to 81 years). All patients were clinically examined by an experienced neurologist at the time of the MRI scan, and the duration of symptoms was evaluated. The scores on the ALS Functional Rating Scale (ALSFRS) [14] and its revised version (ALSFRS-R) [15] were recorded. The patients were classified using both El Escorial and Awaji criteria.

An age- and sex-matched control group of 15 healthy individuals was acquired (four female, eleven male, mean age 54 years, range 36 to 67 years). Similarly to the patients, exclusion criteria were neurodegenerative diseases, spinal canal stenosis and/or any intramedullary lesion on MRI, relevant contraindications for MRI and relevant motion artifacts.

**Fig 1:**
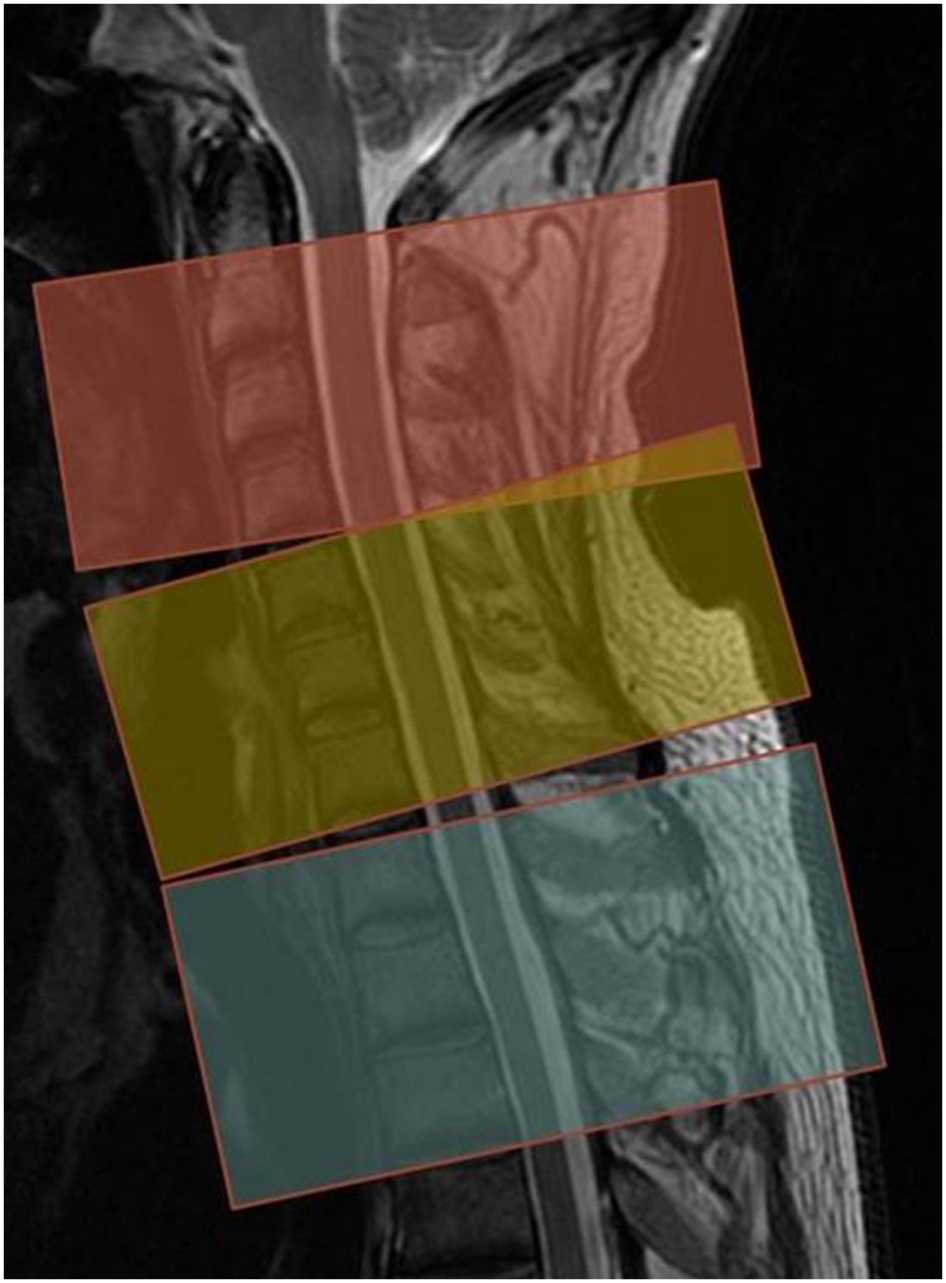
DTI planning. Sagittal T2-weighted image used for planning the axial sequences. The different-coloured blocks demonstrate the three levels of the axial DTI blocks.

The MRI examinations were performed at a 3.0-Tesla-MRI scanner (Signa HDx 3T, GE Healthcare, Milwaukee, Wisconsin, USA) using a 16-channel spine coil. The protocol consisted of a sagittal T2-weighted sequence (TR/TE 3247/114 ms; 3mm slice thickness), a sagittal T1-weighted sequence (TR/TE 795/16ms; 3mm slice thickness) and an axial T2-weighted sequence (TR/TE 3436/103ms; slice thickness 3mm). The first block was acquired from C2 to C4, the second from C5 to C7, and the third from Th1 to Th3. DTI was acquired as a spin-echo single-shot echo-planar sequence (SE/EPI) with diffusion gradients in 15 spatial directions and the following parameters: TR/TE 5000/87ms, field of view 120 x 120mm, matrix 64 x 64, b value 700 sec/mm^2^, slice thickness 5mm, no interslice gap, six averages. Three acquisitions with 12 axial sections were acquired parallel to the intervertebral disc space at the levels C2-C4, C5-C7 and Th1-Th3 (Fig 1). The overall scanning time for the three DTI acquisitions was 16:45 min. The diffusion-tensor imaging data were analysed using FuncTool software (GE Healthcare, Buc, France) at a commercially available workstation (AW 4.2; GE Healthcare, Buc, France). Axial fractional anisotropy (FA) maps were generated and overlaid with the T2-weighted images to correctly detect the spinal cord. A motion correction algorithm was applied to correct for the spine motion and image distortion due to eddy current artefacts.

**Fig 2:**
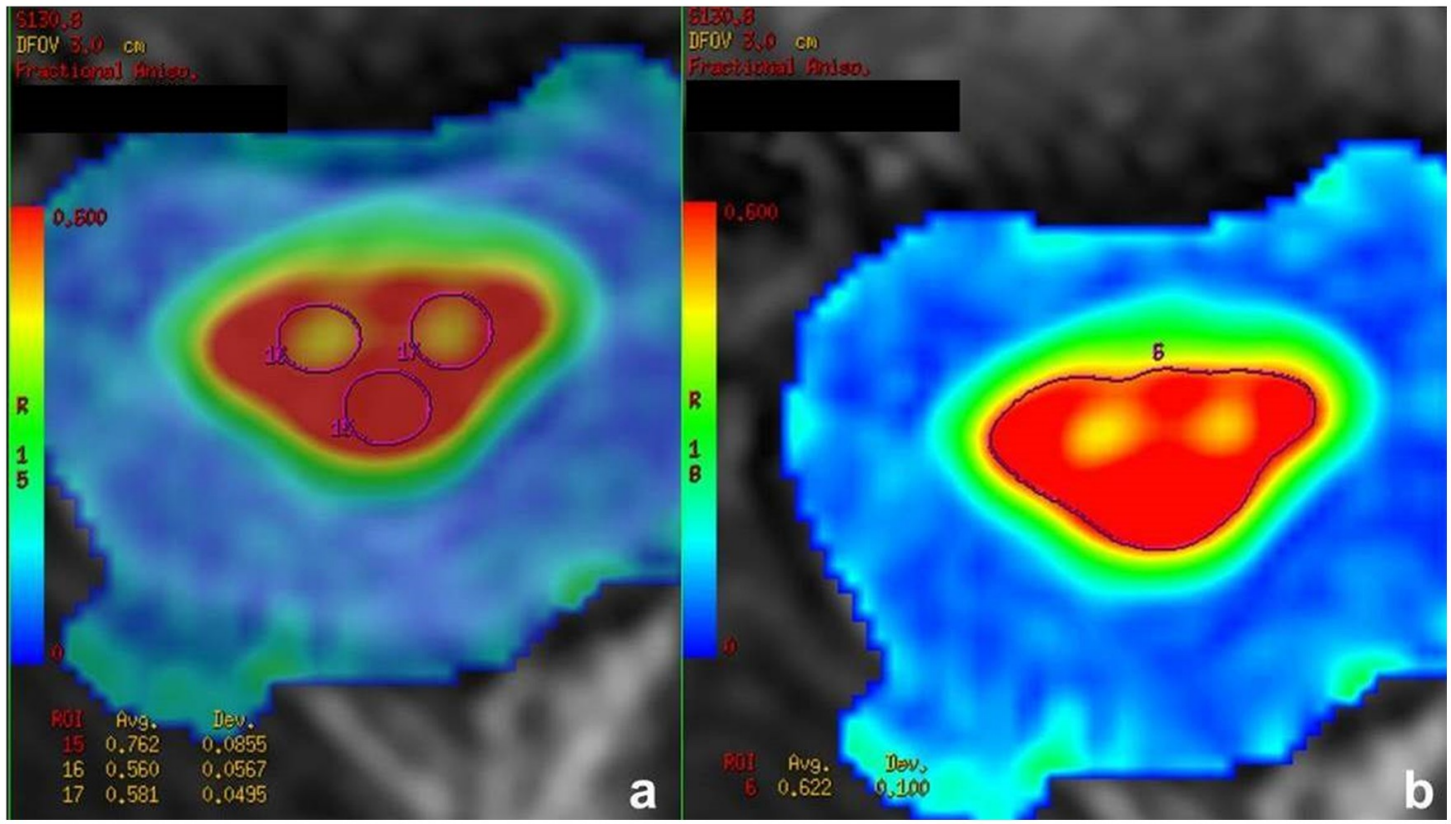
ROI placement. Placement of the measurement ROIs on the axial FA maps. The anterolateral and posterior ROIs are depicted in image a, the cross section ROI in image b.

To measure the FA values, four ROIs were placed manually on each slice. Two ROIs were placed in the right and left anterolateral region of the spinal cord, including the corticospinal tract and anterior horn cells. Furthermore, one ROI was drawn in the median posterior part of the cord, thus comprising the sensory tracts. Finally, one ROI was placed over the whole cross section of the spinal cord (Fig 2). Due to the smaller cross section of the thoracic spinal cord, no valid measurements of separate smaller ROIs were possible at this level. As a result, only the ROI including the whole cross section was applied at the level Th1-Th3. The ROI measurements were performed for each slice of the three DTI blocks. Overall, 108 ROIs were applied for each subject. The mean values for the anterolateral, posterior and cross section ROI were calculated for each subject and each of the three levels. ROIs were drawn in consensus by two radiologists with several years of experience in neuroradiology and DTI acquisition/evaluation. The evaluators were blinded to the clinical status of the patients. In one patient, only the C5-C7 ROI could be evaluated while the other measurements had to be excluded due to motion artifacts.

The statistical calculations were performed with SPSS V.14 software (SPSS, Chicago, Ill). 1). Data were tested for normal distribution using the Kolmogorov-Smirnoff test. As the data was mostly not normally distributed, the non-parametric Mann-Whitney-U-Test was applied to compare the two study groups. A p-value < 0.05 was considered to be statistically significant. The Spearman rank correlation coefficient (Spearman’s ρ) was calculated for correlations between FA values and clinical parameters (ALSFRS/ALSFRS-R scores).

## Results

### Patient classification

According to the El-Escorial classification of 2000 [16], five patients had definite ALS, five patients had laboratory supported probable ALS, and four patients had clinically probable ALS. Applying the Awaji criteria, the patients were classified as having clinically probable ALS in nine cases, and clinically definite ALS in five cases. Retrospectively, within the course, all patients met the El Escorial criteria in its latest revision of 2015 [16].

In the clinical examination at the time of the MRI, six patients presented lower motor neuron signs with upper and lower limb pareses, six patients had only upper limb pareses and two patients had only lower limb pareses. Twelve patients presented with clinical signs of upper motor neuron involvement, concerning both upper and lower extremities in six patients, only the upper extremities in two patients and only the lower extremities in four patients. Bulbar symptoms and impaired respiration were found in three cases each. None of the patients showed clinical signs of cognitive impairment. The median ALSFRS and ALSFRS-R scores were 35.5 (range 16–38) and 43.5 (range 24–46), respectively. The median duration of the ALS symptoms was 20 months (range 5–108 months).

### DTI MRI Data

**Fig 3:**
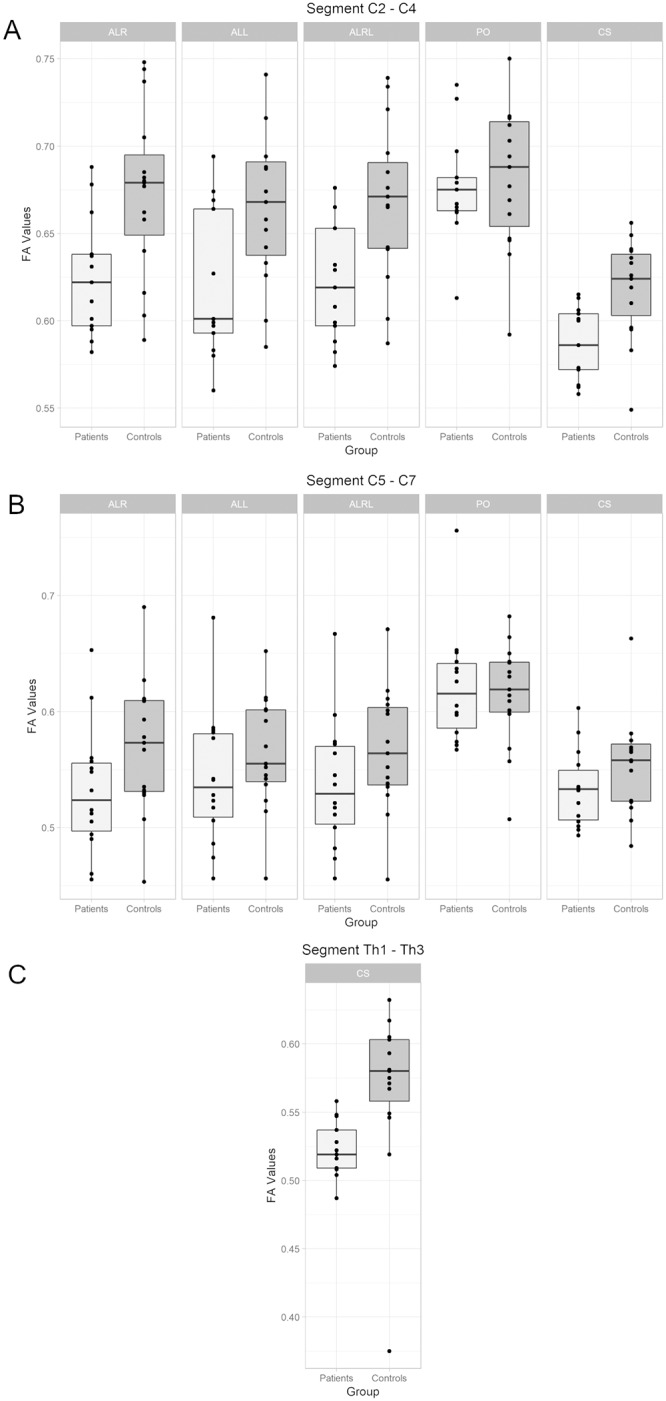
Fractional anisotropy measurements. Boxplots depicting the FA measurement results at the C2-C4 level (Fig 3A), C5-C7 level (Fig 3B) and Th1-Th3 level (Fig 3C). No statistically significant correlation was found between the FA values of the different anatomical locations and ALSFRS/ALSFRS-R scores (p-values ≥ 0.108).

The FA values of both anterolateral ROIs and the cross section ROI were significantly reduced in the ALS patient group in comparison to the control group at the level C2-C4. The FA of the cross section at the Th1-Th3 level was also significantly reduced in the ALS group. There were no significant differences at the C5-C7 level, although there was a trend towards a significantly reduced FA in the anterolateral ROIs of ALS patients at this level. No significant differences or statistical trends were found in the posterior ROIs at the C2-C4 and C5-C7 levels. The absolute measurement values and the results of the statistical analysis are presented in Table 1 and Fig 3.

**Table 1:** Fractional anisotropy measurements.

	Patients	Controls	P-Value
**ALR C2-C4**	.622 (.596–.650)	.679 (.640–.705)	.007
**ALL C2-C4**	.601 (.588–.666)	.668 (.633–.694)	.022
**ALRL C2-C4**	.619 (.592–.659)	.671 (.641–.670)	.010
**PO C2-C4**	.675 (.662–.690)	.688 (.647–.716)	.618
**CS C2-C4**	.586 (.567–.605)	.624 (.596 –.640)	.003
**ALR C5-C7**	.523 (.493–.557)	.573 (.530–.610)	.077
**ALL C5-C7**	.534 (.501–.583)	.555 (.537–.602)	.134
**ALRL C5-C7**	.529 (.496–.572)	.564 (.535–.606)	.093
**PO C5-C7**	.616 (.580–.647)	.619 (.598–.645)	.949
**CS C5-C7**	.533 (.504–557)	.558 (.522–.575)	.146
**CS TH1-TH3**	.519 (.508–.542)	.580 (.549–.603)	< .001

Measurement values are presented as median and interquartile range (in brackets). The p-value refers to the comparison of the two groups by the non-parametric Mann-Whitney-U-Test.

FA, fractional anisotropy; ALR, right anterolateral region of interest (ROI); ALL, left anterolateral ROI; ALRL, right and left anterolateral ROI (taken together); PO, posterior ROI (PO); CS, cross section ROI.

## Discussion

DTI—MRI is based on the measurement of the three-dimensional diffusivity of water. In CNS tissue, molecular diffusion is anisotropic, i.e. it is restricted and directed by different “barriers” such as cell membranes, axons and particularly myelin sheaths. The degree of anisotropy can be quantified by the DTI parameter FA. Pathological processes which impair the microstructure of CNS tissue (such as inflammation, demyelination as well as axonal degeneration) will alter the anisotropy of water diffusion. Thus, FA has been used as a marker of microstructural integrity. In a recent meta-analysis on modern spinal cord imaging, FA proved to be the most useful DTI parameter regarding the potential as a biomarker for disease severity and the measurement of the degree of tissue injury for different pathologies (such as cervical spondylotic myelopathy, multiple sclerosis, neuromyelitis optica and ALS [17]). However, the evidence level regarding the latter is still low [17].

Our study demonstrated significantly reduced FA values in ALS patients compared to the control group, which affirms the potential of DTI as a diagnostic tool for ALS. Previous studies have shown reduced FA in ALS patients, using sagittal [18], coronal [19] or axial [20] sequences and measuring an average fractional anisotropy of the whole cervical cord. By measurements of specific ROIs on axial sections at different levels of the cervical and upper thoracic spine, we attempted a detailed look at spinal cord pathology. The anterolateral ROIs were chosen to include the corticospinal tract and anterior horn cells, thus comprising both the upper and lower motor neuron. Additionally, a posterior ROI representing sensory tracts as well as the whole cross section were assessed. Budrewicz et al. [21] and Cohen-Adad et al [22] also performed FA quantifications with axial ROIs and found disparities between ALS patients and healthy controls in both anterolateral and posterior columns. Our findings diverge from these results, as we found significant differences in ROIs representing the motor system, but not in those representing sensory tracts. Our results are consistent with the known pathophysiology of ALS as primarily a motor neuron disease, and are in line with an animal study which showed DTI changes in motor tracts but not in sensory pathways in a mouse model [23].

Regarding the sagittal distribution of tissue damage, our results also differ from previous studies. The most severe changes as measured by DTI have so far been reported for the lower cervical spine [19, 21, 22]. This has been interpreted as supporting the “dying back” theory, i.e. a retrograde spread of axonal damage in the corticospinal tract in ALS patients. We did find lower absolute FA values in the C5-C7 segments compared to the C2-C4 segments, but this was the case in both patient and control group. Significant differences between the groups were found in the upper cervical and the upper thoracic levels, but not in the lower cervical cord. While we believe that the distribution of microstructural disintegrity could be influenced by the direction of neuronal degeneration, it is probably also influenced by the heterogeneity of clinical phenotypes in ALS. In the current patient group, for example, there were five patients who had no or very mild distal upper extremity paresis. In addition to a limited sample size, this could explain why the FA values at the lower cervical segments did not reach statistical significance.

One would expect that FA values correlate with the severity of the disease. In our study, however, we did not find a statistically significant correlation of clinical parameters and FA metrics. So we could not corroborate the potential of FA as a biomarker. However, this could be due to the relatively small cohort size.

There were several limitations of the present study. Firstly, as mentioned above, the number of patients was relatively low, possibly precluding further significant results. Secondly, the ROIs were drawn manually, which could limit the reproducibility of the measured values. Thirdly, some of the patients were included early in the disease course (having a probable, yet not definite diagnosis of ALS, but met the 2015 El Escorial criteria within the further course) when the damage of the motor tracts was still mild. However, on the basis of a larger age-matched and muscle type-specific dataset this could allow corroborating the diagnosis even in early disease stages.

### Conclusion

With the finding of reduced FA in the motor tracts of ALS patients, we present further evidence for the potential of DTI in ALS diagnostics. In the future, DTI could be used both as a diagnostic criterion, and as a biomarker for disease progression and possibly treatment effects. On closer inspection, however, our findings and the results of previous studies differ to a certain degree. This is likely due to clinically different patient groups, but also to different methods of image acquisition and measuring. Consequently, larger studies which compare different DTI methods and relate them to clinical parameters are needed. Hereby, DTI acquisition, quantification, and interpretation should become as standardised as possible, thus significantly increasing its diagnostic value.

## Supporting information

S1 Table Fractional anisotropy measurements and clinical scores. (XLSX)
